# Doppler study of portal vein and renal venous velocity predict the appropriate fluid response to diuretic in ICU: a prospective observational echocardiographic evaluation

**DOI:** 10.1186/s13054-022-04180-0

**Published:** 2022-10-05

**Authors:** Pierre-Grégoire Guinot, Pierre-Alain Bahr, Stefan Andrei, Bogdan A. Popescu, Vincenza Caruso, Paul-Michel Mertes, Vivien Berthoud, Maxime Nguyen, Belaid Bouhemad

**Affiliations:** 1 Department of Anaesthesiology and Critical Care Medicine, Dijon University Medical Centre, 21000 Dijon, France; 2 University of Burgundy and Franche-Comté, LNC UMR1231, 21000 Dijon, France; 3 Department of Anaesthesiology and Critical Care Medicine, University of Medicine and Pharmacy “Carol Davila”, Bucharest, Romania; 4 Euroecolab, Emergency Institute for Cardiovascular Diseases “Prof Dr C C Iliescu”, Bucharest, Romania; 5 Department of Anaesthesiology and Critical Care Medicine, Strasbourg University Medical Centre, Strasbourg, France

**Keywords:** ICU, POCUS, Echography, Congestion, Venous, Cardiac, Portal pulsatility index

## Abstract

**Background:**

Fluid overload and venous congestion are associated with morbi-mortality in the ICU (intensive care unit). Administration of diuretics to correct the fluid balance is common, although there is no strong relationship between the consequent fluid loss and clinical improvement. The aim of the study was to evaluate the ability of the portal pulsatility index, the renal venous impedance index, and the VEXUS score (venous ultrasound congestion score) to predict appropriate diuretic-induced fluid depletion.

**Methods:**

The study had a prospective, observational, single-center observational design and was conducted in a university-affiliated medico-surgical ICU. Adult patients for whom the clinician decided to introduce loop diuretic treatment were included. Hemodynamic and ultrasound measurements (including the portal pulsatility index, renal venous impedance index and VEXUS score) were performed at inclusion and 2 hours after the initiation of the diuretics. The patients’ characteristics were noted at inclusion, 24 h later, and at ICU discharge. The appropriate diuretic-induced fluid depletion was defined by a congestive score lower than 3 after diuretic fluid depletion. The congestive score included clinical and biological parameters of congestion.

**Results:**

Eighty-one patients were included, and 43 (53%) patients presented with clinically significant congestion score at inclusion. Thirty-four patients (42%) had an appropriate response to diuretic-induced fluid depletion. None of the left- and right-sided echocardiographic parameters differed between the two groups. The baseline portal pulsatility index was the best predictor of appropriate response to diuretic-induced fluid depletion (AUC = 0.80, CI_95%_:0.70–0.92, *p* = 0.001), followed by the renal venous impedance index (AUC = 0.72, CI_95%_ 0.61–0.84, *p* = 0.001). The baseline VEXUS score (AUC of 0.66 CI_95%_ 0.53–0.79, *p* = 0.012) was poorly predictive of appropriate response to diuretic-induced fluid depletion.

**Conclusion:**

The portal pulsatility index and the renal venous impedance index were predictive of the appropriate response to diuretic-induced fluid depletion in ICU patients. The portal pulsatility index should be evaluated in future randomized studies.

**Supplementary Information:**

The online version contains supplementary material available at 10.1186/s13054-022-04180-0.

## Introduction

In recent years, there has been a growing interest in the diagnosis and treatment of fluid overload and venous congestion [1, 2]. The first studies from cardiology and cardiac surgical area demonstrated an association between heart failure (acute or chronic), venous congestion, fluid overload and organ dysfunction [3–5]. Fluid overload implies peripheral edema but also could result in pulmonary edema and venous congestion [6]. Fluid overload alters tissue perfusion in relation to venous congestion and/or tissue edema. Subsequently, several studies have shown an association between fluid overload and morbi-mortality in ICU (intensive care unit) [1, 6].

Physicians empirically use diuretics, mostly loop diuretics [7], to correct the fluid balance, improve diuresis and treat venous congestion. An expert position statement provided recommendations on diuretics treatment [7]. Studies have demonstrated that there is no strong relationship between the net fluid loss during treatment of venous congestion and further clinical improvement [8]. Solely applying a restrictive or liberal fluid strategy may not be the optimal strategy, because at the bedside, it is difficult to discriminate between patients with organ dysfunction in relation to venous congestion and fluid overload from those with cumulative fluid balance. Also, the natriuretic response to the diuretic dose test may not always predict appropriate decongestion because of intra- and interpatient variability [9]. These observations suggest that response to diuretics should not probably be evaluated only in terms of natriuresis and net fluid loss but also in terms of effects on reproducible parameters.

Recently, several ultrasound indices of venous congestion have been studied in the context of heart failure and/or cardio-renal syndrome [3, 5, 10]. Measurement of portal pulsatility index, renal venous impedance, and/or the construction of the VEXUS score (venous ultrasound congestion score) are associated with venous congestion, occurrence of acute renal failure, and clinical prognosis of patients [3, 11]. Of these parameters, the pulsatility portal index and the venous renal flow are two promising parameters that have been associated with venous congestion and fluid overload [12–14]. The pulsatility portal index has been demonstrated to increase with fluid expansion when patients were unable to increase their cardiac output (i.e., fluid unresponsive) [15]. In the same way, the portal pulsatility index increase with the increase in positive end-expiratory pressure and central venous pressure [16]. Apart from these observations, the pulsatility portal index may reflect venous congestion in relation to volemia. Case reports have suggested the ability of portal pulsatility index and renal venous impedance to be associated with the clinical response to diuretic fluid depletion [12]. Currently, there are no studies in the ICU on the usefulness of the portal pulsatility index in identifying patients that will respond to diuretic fluid depletion.

The primary objective was to evaluate the ability of the portal pulsatility index to detect appropriate response to diuretic-induced fluid depletion. The secondary objectives were to evaluate the value of the renal venous impedance index and the VEXUS score to detect appropriate response to diuretic-induced fluid depletion.

## Methods

### Patients

We performed a prospective, observational, single-center study in a cardiovascular medico-surgical ICU of a tertiary university medical center (Dijon, France) between 2019 and 2020. This study was approved by the French Comité de Protection des Personnes (2018/7148). All patients received a written informed letter and gave consent to participate. The study was performed in accordance with the ethical standards laid down in the 1964 Declaration of Helsinki.

We consecutively included all patients with age > 18 years, presence of clinical signs of fluid overload, absence of fluid responsiveness assessed by an increase in stroke volume following passing leg raising; and for whom the clinician decided to introduce loop diuretic treatment for several days. The decision to initiate loop diuretic treatment was left to the treating physician and was noted from medical data. The main non-inclusion criteria were prior diuretic treatment during the ICU stays, permanent atrial fibrillation, veno-venous hemofiltration or dialysis, and patients with unstable shock (variation of blood pressure > 10% despite hemodynamic treatment and/or need to increase hemodynamic support).

### Echocardiographic measurements

Transthoracic echocardiography was performed by an experienced physician (advanced level training) using a Philips Envisor ultrasound system (Affinity ultrasound system Philips Medical System, Suresnes, France), according to current guidelines [17, 18]. The echocardiographic parameters were calculated as the average of five measurements (regardless of the respiratory cycle). Data were acquired and stored for later analysis. The images were reviewed offline by an experienced operator blinded to the study outcomes. The attending physician was unaware of the results of the ultrasound examination. The LVEF (left ventricular ejection fraction) was measured using Simpson’s biplane method. The diameter of the LVOT (left ventricular outflow tract) was measured in a parasternal long-axis view upon patient inclusion. The AVA (aortic valve area, in cm^2^) was calculated as *π* × LVOT^2^/4. The VTIAo (aortic velocity–time integral) was measured by PW (pulsed wave) Doppler and a five-chamber apical view. SV (Stroke volume, in mL) was calculated as VTIAo × AVA, and CO (cardiac output, in L. min^-1^) as SV × HR (heart rate). In patients with TR (tricuspid regurgitation), peak TR velocity was measured by CW (continuous wave) Doppler, and the right ventricle–right atrium pressure gradient was calculated. The right ventricular systolic function was assessed by measuring TAPSE (tricuspid annular plane systolic excursion) and RVFAC (right ventricular fractional area change). The hepatic venous flow was recorded from the subcostal window.

### Portal, hepatic and renal Doppler measurements

The HV-S (supra-hepatic vein systolic) and HV-D (supra-hepatic vein diastolic) velocities were measured, and the S/D (systolic/diastolic) ratio was calculated. With the patient in the supine position, the diameter of the inferior vena cava was measured in the subcostal view at 1 cm from its junction with the right atrium. The maximum and minimum diameters of the inferior vena cava were measured, and the percentage of change in diameter was calculated.

The PI (portal pulsatility index) was assessed by pulsed-wave Doppler evaluation of the portal vein in the liver hilum [16]. The Vmax (maximum velocity) and Vmin (minimum velocity) of the portal vein were measured in PW Doppler mode. The portal PI was calculated using the following formula: PI = (*V*max − *V*min)/(*V*max). We calculated intra- and inter-observer reproducibility. Intra and inter-observer reproducibility was 10% (4–16) and 15% (11–28), respectively.

RRI (Renal resistive index) and RVI (renal venous impedance index) were measured using a transparietal 5 MHz pulsed-wave Doppler probe. Doppler measurements were performed in the interlobar arteries/veins of the upper, median, and lower segments of each kidney [3, 19]. For each artery, RRI was calculated as: RRI = (peak systolic velocity − end-diastolic velocity)/peak systolic velocity. For each vein, RVI was calculated as: RVI = (peak systolic velocity − end-diastolic velocity)/peak systolic velocity. All values were the average of 3 measurements. RRI and RVI then were calculated as the average RRI and RVI for each kidney. Intra and inter-observer reproducibility of RI and RVI is 0 ± 9% and 3 ± 12%, and VII, 4 ± 13% and 5 ± 12%, respectively [20]. In addition, Doppler waveforms of RVI were divided into 5 flow patterns: continuous, continuous pulsatile, biphasic discontinuous (systolic wave > diastolic wave), biphasic discontinuous (diastolic wave > systolic wave), and monophasic discontinuous [21].

### Definitions and scores

Based on the literature [4, 22], we constructed a congestion score based on clinical indicator and biomarkers of congestion: pulmonary rales/crackles (graded between 0 (no), 1 (< 50% of lung) and 2 (> 50% of lung)), peripheral edema (graded between 0 (no), 1 (ankle), 2 (leg) and 3 (body)), B-lines and/or lung ‘comets’ (graded between 0 (no), 1 (more than 2 area) and 2 (diffuse)), and/or pleural effusion (graded between 0 (no), 1 (unilateral) and 2 (bilateral)) on lung ultrasound, and NT-proBNP (N-terminal pro B-type natriuretic peptide) value over 1500 pg mL^−1^ [4, 22]. The positive fluid balance was not a criterion in the clinical congestion score calculation. The congestion score ranged from 0 to 10, and a patient with a score ≥ 3 was considered as having significant clinical congestion [4, 22]. Then, appropriate diuretic-induced fluid depletion was considered with a score lower than 3 at the end of the study for patients who had significant clinical congestion at baseline [22]. Patients with baseline congestion score lower than 3 or who did not lower the congestion score with diuretic were considered as the control group.

The VEXUS score was calculated as previously described [11]. The percentage of fluid overload adjusted for body weight was calculated as ((total fluid in − total fluid out)/admission body weight × 100) [23].

### Study protocol

Patients were followed during all their ICU stay. Based on an expert opinion statement [7] that suggests assessing the response to diuretic treatment 2 hours after its introduction by measuring natriuresis and diuresis, we performed hemodynamic and echographic measurements at these time points to compare them with natriuresis/diuresis.

Clinical examination, lung ultrasound, urine output, diuretic, vasoactive treatments, NT-proBNP, and blood creatinine were recorded at inclusion, 24 h later, and at ICU discharge. Cumulative fluid balance and cumulative percentage of fluid overload were calculated at inclusion, 24 h later, and at ICU discharge. The VEXUS score and the congestion score were calculated at inclusion and 2 h later.

The primary endpoint was the appropriate diuretic-induced fluid depletion on the last day of ICU [22].

### Statistical analysis

We calculated that a sample size of 74 patients would be sufficient to demonstrate that the pulsatility portal index can predict the response to diuretic with an AUC (area under the curve) between 0.7 and 0.8, a power of 90%, an alpha risk of 0.05 and a beta risk of 0.1. We included up to 81 patients to take into account the risk of incomplete data. Two groups of patients were compared: patients with and without positive clinical response to diuretic-induced fluid depletion. Normality was assessed using the Shapiro–Wilk test. Accordingly, quantitative data are presented as medians (interquartile range) or as mean (standard deviation). Qualitative data are presented as frequency and percentage. Quantitative and qualitative variables were analyzed by using the Mann–Whitney test, the Student test, the paired Student test, or the Wilcoxon test, as appropriate. Bonferroni post hoc corrections were used to assess statistical significance, as appropriate. To assess the relationship between echographic parameters and the positive clinical response to diuretic-induced fluid depletion, we performed a ROC (Receiver Operating Characteristic) curve with calculating the AUC. The method described by DeLong et al. was used to compare the areas under the ROC curve associated with the variables [24]. The cutoff value was chosen with the highest sensitivity and the highest specificity. Sensitivity, specificity, positive predictive value, negative predictive value, and their 95% confidence intervals were calculated for the best cutoff values. The associations between cardiovascular variables and appropriate diuretic-induced fluid depletion response were assessed using a univariate logistic regression model. Variables with a *p* value of 0.10 (cardiac index, portal pulsatility, renal venous index pattern, stroke volume change following passive leg raising, and left ventricular E/A ratio) were then included in a multivariate logistic model with a backward selection procedure. All reported probability values were two-tailed, and a *p* value ≤ 0.05 was considered statistically significant. All analyses were performed using R software version 3.4.4 (R Foundation for Statistical Computing, Austria).

## Results

### Population

**Fig. 1:**
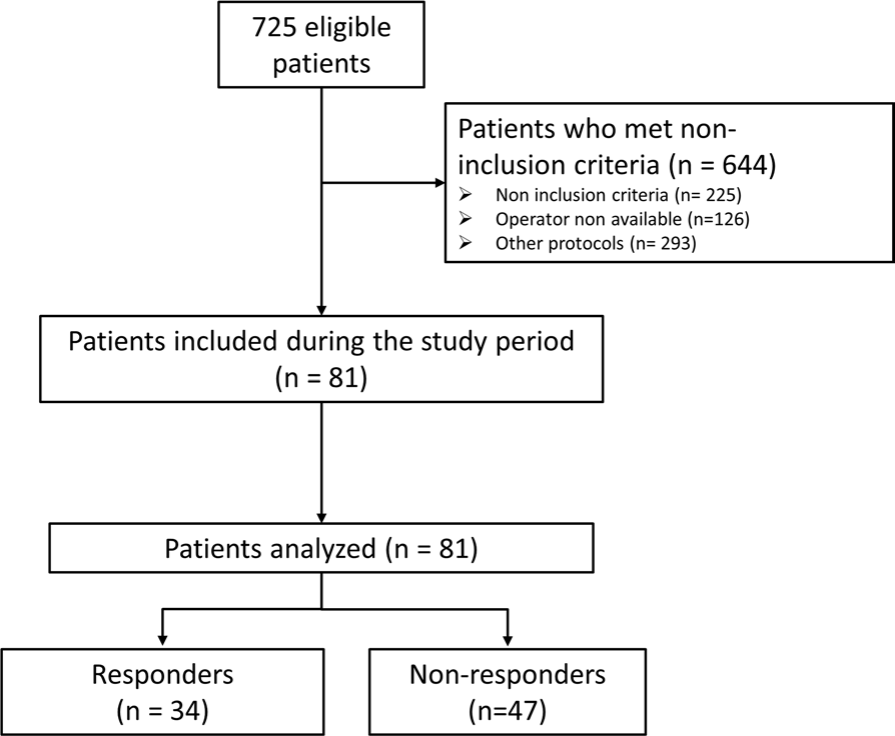
Study flowchart. No patients were excluded

Eighty-one patients were included and analyzed (Fig. 1). In the overall study population, the mean age was 68 ± 11 years (males: *n* = 51 (63%) and the median SAPS II (Simplified Acute Physiology Score II) was 46 ± 18 (Table 1). Diuretics were given for oliguria (*n* = 25), positive fluid balance (*n* = 63) and congestion (*n* = 18). Of the 81 patients, the median score was 3 (2–4), and 43 patients (53%) had a congestive score over 3 (Table 2). The median change of congestion score between baseline and ICU discharge was − 1 (− 3–0). Nine patients (10%) died, and they were included in the control group. Thirty-four patients (42%) have an appropriate response to diuretic-induced fluid depletion on the last day of ICU (i.e., a decrease in congestion score).

**Table 1:** Baseline characteristics of the cohort

Age (years), mean (SD)	68 (11)
Women, *n* (%)	30 (37%)
Body mass index (kg m^−2^), mean (SD)	27 (6)
SAPS II, mean (SD)	46 (18)
*Medical history, n (%)*	
Chronic high blood pressure	54 (67%)
Diabetes (insulin dependent and non-insulin dependent)	27 (33%)
Cardiopathy	
Ischemic	37 (46%)
Valvular (mitral, aortic)	32 (39%)
Chronic renal failure	17 (21%)
Estimated glomerular filtration rate (ml min^−1^ 1.73 m^−2^), mean (SD)	77 (22)
*Admission to ICU, n (%)*	
Medical/surgical	32/49
Cardiac surgery (CABG and/or valvular)	49 (60%)
Septic shock	13 (16%)
Cardiogenic shock	12 (15%)
Other (hemorrhagic shock, polytrauma, stroke, subarachnoid hemorrhage)	7 (9%)
Mechanical ventilation during ICU stays, n (%)	72 (89%)
Fluid overload at inclusion (%), median (IQR)	4.5 (0.8–6.5)
ICU length of stay (days), median (IQR)	5 (3–10)

*SD*, standard deviation; *SAPS II*, simplified acute physiology score II; *ICU*, intensive care unit; *CABG*, coronary artery bypass graft; 25–75% *IQR*, interquartile range

**Table 2:** Evolution of the cohort during the study period

	Baseline	24 h	ICU discharge
Norepinephrine, *n* (%)	21 (27%)	14 (17%)	0*
Dobutamine, *n* (%)	14 (17%)	3 (4%)*	0*
*Diuresis, median (IQR)*			
6 h (ml)	238 (170–348)	650 (329–948)*	470 (342–650)
6 h ml kg^−1^ h^−1^	0.5 (0.4–0.8)	1.4 (0.6–2.1)*	1 (0.8–1.5)
24 h fluid balance (ml), median (IQR)	989 (171–1589)	− 660 (− 1451–263)	− 1110 (− 2384 to − 16)*
*Congestion criteria, n (%)*			
Pulmonary rales/crackles	31 (39%)	10 (12%)	4 (5%)
Peripheral edema	43 (53%)	30 (37%)	11 (14%)
B-lines, lung comets	18 (22%)	11 (14%)	5 (6%)
Pleural effusion	20 (25%)	19 (23%)	14 (17%)
Congestion score, median (IQR)	3 (2–4)	2 (1–3)	1 (0–2)*
NT-proBNP, (pg ml^−1^), median (IQR)	2905 (929–5818)	2693 (1191–5194)	2175 (845–5854)*
Weight (Kg), mean (SD)	76 (17)	NA	75 (15)

A post hoc Bonferroni correction was applied

*SD*, standard deviation, *IQR*, interquartile range, *ICU*, intensive care unit, *NT-proBNP*, N-terminal (NT)-pro hormone brain natriuretic peptide, *NA*, not available

*p < 0.05 with baseline

The diuretic bolus dose (40 mg (23–48) versus 40 mg (40–80), *p* = 0.642), the total daily dose (70 mg (35–134) versus 60 mg (40–120), *p* = 0.311), and the total fluid balance at day one (− 505 ml (− 1465–385) versus − 770 ml (− 1451–155), *p* = 0.458) did not differ between appropriate diuretic-induced fluid depletion group and control group.

### Comparison between patients with and without positive clinical response to diuretic-induced fluid depletion

Hemodynamic and echocardiographic parameters at baseline and at 2 hours for both groups of patients are given in Table 3. Two-hour diuresis and 2-hour natriuresis spots did not differ between the two groups: 558 ml (319) versus 683 ml (354), *p *= 0.137, and 91 mmol l^−1^ (33) versus 93 mmol l^−1^ (36), *p* = 0.87. The VEXUS score was higher for patients with diuretic-induced fluid depletion than for the control group (2 (0–3) vs 0 (0–2), *p* = 0.03).

**Table 3:** Hemodynamic, portal, hepatic, and renal Doppler measurements and VEXUS score according to congestive response to diuretic treatment

	Baseline	Two hours
*Hemodynamic parameters at inclusion*		
Heart rate (bpm), mean (SD)		
Appropriate fluid depletion	83 (16)	82 (14)
Control	80 (18)	82 (16)
Mean arterial pressure (mmHg), mean (SD)		
Appropriate fluid depletion	80 (11)^$^	79 (12)
Control	85 (13)	85 (13)
Central venous pressure (mmHg), mean (SD)		
Appropriate fluid depletion	14 (4)	12 (4)
Control	13 (5)	12 (5)
Cardiac index (l min^−1^ m^−2^), mean (SD)		
Appropriate fluid depletion	2.7 (0.9)^$^	2.6 (1)
Control	2.5 (0.4)	2.6 (0.8)
Diuresis (ml kg^−1^ h^−1^), median (IQR)		
Appropriate fluid depletion	0.5 (0.4–0.7)	1.6 (0.8–2.7)*
Control	0.6 (0.3–0.8)	1.7 (0.9–2.8)*
*Portal, hepatic, and renal variables*		
IVC diameter (cm), mean (SD)		
Appropriate fluid depletion	2.2 (0.5)	2.1 (0.4)
Control	2.2 (0.4)	2.1 (0.4)
S/D sus-hepatic wave ratio, median (IQR)		
Appropriate fluid depletion	0.6 (− 0.6–1.1)	0.5 (0.4–1.1)
Control	0.9 (0.6–1.1)	1 (0.6–1.4)
Mean portal velocity (cm s^−1^), mean (SD)		
Appropriate fluid depletion	19 (6)	19 (6)
Control	21 (6)	21 5)
Portal pulsatility index (%), median (IQR)		
Appropriate fluid depletion	45 (30–68)^$^	38 (21–48)^$,^*
Control	27 (22–35)	24 (12–31)*
Renal venous impedance index, median (IQR)		
Appropriate fluid depletion	− 0.05 (− 0.3–0.4)	− 0.15 (− 0.3–0.8)^$^
Control	0.20 (0.03–0.38)	0.18 (0.03–0.28)
Renal venous impedance pattern, n (%)		
Continuous		
Appropriate fluid depletion	1 (1%)	2 (2%)
Control	5 (6%)	5 (6%)
Pulsatile		
Appropriate fluid depletion	7 (9%)	3 (4%)
Control	18 (22%)	17 (21%)
Biphasic discontinuous (S wave > D wave)		
Appropriate fluid depletion	6 (7%)	6 (7%)
Control	15 (19%)	16 (20%)
Biphasic discontinuous (D wave > S wave)		
Appropriate fluid depletion	16 (20%)	20 (25%)
Control	9 (11%)	9 (11%)
Monophasic discontinuous		
Appropriate fluid depletion	4 (5%)	3 (4%)
Control	0	0
*VEXUS score, n (%)*		
Grade 0		
Appropriate fluid depletion	10 (12%)	12 (15%)
Control	16 (20%)	23 (28%)
Grade 1		
Appropriate fluid depletion	4 (5%)	1 (1%)
Control	10 (12%)	11 (14%)
Grade 2		
Appropriate fluid depletion	5 (6%)	9 (11%)
Control	16 (20%)	12 (12%)
Grade 3		
Appropriate fluid depletion	15 (19%)	12 (15%)
Control	5 (6%)	3 (4%)

Diuresis refers to previous 2 h of baseline, and following 2 hours after diuretic administration

*IVC*, inferior vena cava diameter, *VTI*, velocity time integral, *S*, systolic, *D*, diastolic, *SD*, standard deviation, *IQR*, interquartile range

^$^*p* < 0.05 comparison between control group and appropriate fluid depletion.*p < 0.05 comparison with baseline. Mean (SD), median (IQR), or n (%), as appropriate

At baseline, the portal pulsatility index was higher in patients with appropriate diuretic-induced fluid depletion (45% (30–68) vs 28% (22–35), *p* = 0.001). The venous impedance pattern was significantly worse in patients with appropriate diuretic-induced fluid depletion (2 (1–2) vs 3 (2–3), *p* = 0.011). The VEXUS score was higher in patients with appropriate diuretic-induced fluid depletion (2 (0–3) vs 1 (0–2), *p* = 0.002). At 2 h, the portal pulsatility index was higher in patients with appropriate diuretic-induced fluid depletion (34% (23–54) vs 24% (12–31), *p* = 0.001). None of the other echocardiographic parameters differed between the two groups, even right-sided echocardiographic parameters and the IVC diameter (Additional file 1: Table S1).

The venous impedance pattern was significantly worse in patients with appropriate diuretic-induced fluid depletion (*p* < 0.05). None of the other echocardiographic parameters differed between the two groups.

### Assessment of the relationship between portal and renal flow parameters, VEXUS score, and the positive clinical response to diuretic-induced fluid depletion

**Fig. 2:**
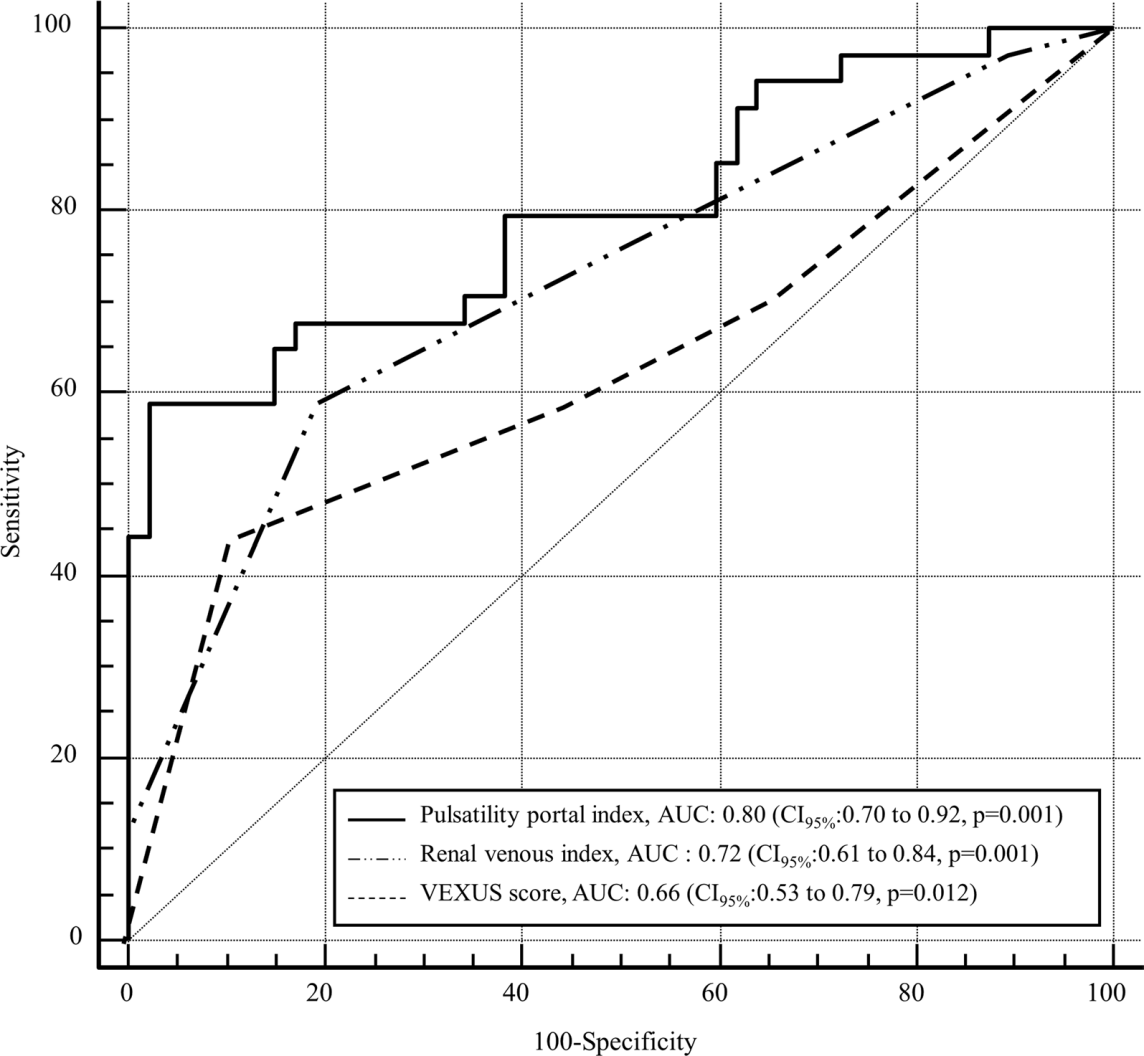
Receiver operating characteristic curves for diuretic congestion improvement prediction by the pulsatility portal index, VEXUS score, and renal venous impedance pattern

At baseline, the portal pulsatility index and venous impedance pattern were associated with the response to diuretic treatment. When analyzed using multivariate logistic regression, portal pulsatility (OR = 20.9 (CI_95%_ 2.8–158.9), *p* = 0.003), cardiac index (OR = 6.9 (CI_95_ 17–28.5), and the impedance venous pattern (OR = 6.3 (CI_95%_ 2.2–18.2) were associated with the diuretic-induced fluid depletion response. The AUC of the portal pulsatility index was 0.80 (CI_95%_ 0.70–0.92, *p* = 0.001) (Table 4 and Fig. 2). The AUC of the impedance venous pattern was 0.72 (CI_95%_ 0.61–0.84, *p* = 0.001) (Table 4). The VEXUS score was also predictive of appropriate diuretic-induced fluid depletion, with an AUC of 0.66 (CI_95%_ 0.53–0.79, *p* = 0.012) (Fig. 2). The AUC of the portal pulsatility index was higher than this of the VEXUS score (*p* = 0.001).

**Table 4:** Diagnostic performance of portal pulsatility index and venous renal flow pattern to predict appropriate diuretic-induced fluid depletion

Variables	AUC (CI_95%_)	Cutoff value	Sensitivity (%)	Specificity (%)	Positive predictive value	Negative predictive value
Portal pulsatility index _*baseline*_	0.80 (CI_95%_: 0.70–0.92)	20%	91 (76–98)	39 (25–54)	52 (46–58)	86 (66–95)
		35%	59 (41–75)	98 (89–100)	95 (74–99)	77 (69–83)
Venous renal flow pattern _*baseline*_	0.72 (CI_95%_: 0.61–0.84)	2	59 (41–75)	81 (67–91)	69 (54–81)	73 (64–81)
		3	12 (3–28)	100 (93–100)	100	61 (58–64)
VEXUS _baseline_	0.66 (CI_95%_: 0.53–0.79)	2	44 (27–62)	89 (77–96)	75 (55–88)	69 (62–75)
Portal pulsatility index _*2 hours*_	0.72 (CI_95%_: 0.63–0.86)	20%	82 (66–95)	41 (27–57)	51 (44–58)	76 (59–88)
		35%	53 (35–70)	89 (76–96)	78 (59–90)	72 (64–79)
Venous renal flow pattern _*2 hours*_	0.77 (CI_95%_: 0.67–0.87)	2	68 (50–83)	81 (67–91)	72 (58–83)	78 (68–85)
		3	9 (2–24)	100 (93–100)	100	60 (58–63)
VEXUS _*2 hours*_	0.67 (CI_95%_: 0.50–0.8)	2	35 (19–53)	93 (82–99)	80 (55–93)	67 (61–72)

*AUC*, area under the curve, *VEXUS score*, venous ultrasound congestion score

*p < 0.05 comparison with baseline. AUC: area under the curve. Cutoff values are presented to offer the best sensitivity or the best specificity

At 2 h, the portal pulsatility index and venous impedance pattern predicted with high specificity appropriate diuretic-induced fluid depletion. The AUC of the portal pulsatility index was 0.72 (CI_95%_:0.63–0.86, *p* = 0.002). The AUC of the impedance venous pattern was 0.77 (CI_95%_:0.67–0.87, *p* = 0.002). The VEXUS score was poorly predictive of appropriate diuretic-induced fluid depletion with an AUC of 0.67 (CI_95%_:0.50–0.8, *p* = 0.002).

## Discussion

This is an exploratory pragmatic study regarding the prediction of response to fluid depletion in ICU patients. Our results may be summarized as follows: (1) patients with an appropriate response to diuretic-induced fluid depletion had high pulsatility portal index and worst renal venous impedance. (2) Right- and left-sided echocardiographic parameters were not associated with an appropriate response to diuretic-induced fluid depletion. (3) Pulsatility portal index and renal venous impedance were the best predictors of appropriate diuretic-induced fluid depletion response to diuretic. The changes in pulsatility portal index and venous renal index predicted the appropriate decongestion with fluid depletion but not response per se to diuretic therapy (amount of fluid). Pulsatility portal index and renal venous impedance are two parameters that may indicate that volume can be mobilized in a congestive state.

Interestingly, in our cohort with patients considered as being in fluid overload, less than half of the cohort had appropriate decongestion, and might truly have needed fluid depletion. The renal response to diuretic (quantified by diuresis and natriuresis) was the same in both groups of patients (responders vs non-responders) and indifferently of appropriate decongestion. This underlines the need to assess the venous congestion more than fluid overload. That also explains the growing interest in the assessment of congestion with different parameters (clinical, echographic, and laboratory) or scores that are associated with clinical outcomes, and that may also predict response to treatment [2, 4, 25]. Our results confirm an association between the pulsatility portal index, the venous renal impedance index, and appropriate decongestion with diuretic fluid depletion. These indices perform better than the VEXUS score for predicting congestion and response to fluid depletion, with the limit that VEXUS score was developed to predict renal failure not decongestion response to treatment [11].

These results can be explained. Congestion is a complex clinical and hemodynamic syndrome that is the consequence of several cardiac and non-cardiac phenomena. In ICU, underlying disease (e.g., acute respiratory distress syndrome, pneumoniae, cirrhosis, etc.), positive pressure ventilation, and therapeutics (fluid therapy, blood transfusion) can further alter cardiovascular homeostasis and increase the prevalence of congestion. Of the factors involved in congestion, volemia is the most difficult parameter to assess in ICU. Parameters usually used to assess volemia evaluate a preload state, and they can be altered by cardiovascular disease, thus they are not good indices of volemia. In the present cohort, patients were fluid unresponsive (i.e., with no significant increase in stroke volume change following PLR (passive leg raising)), and they had frequently right and left-sided echocardiographic parameters of heart failure (alteration of LVEF/RVFAC, increased IVC diameter…). But none of these parameters were associated with an appropriate diuretic-induced fluid depletion response. Interestingly, our findings support a recent consensus paper that proposes to analyze signs of venous stasis to determine fluid status [26].

The inferior vena cava diameter and the supra-hepatic venous flow are two parameters that may better reflect the severity of the underlying cardiovascular homeostasis than patient volemia [5]. The inferior vena cava dilatation is often present in right heart failure or pulmonary hypertension and has been associated with renal function impairment [5]. One daily question remains: does dilatation of the inferior vena cava reflect volemia or heart failure or both? In an elegant case series, Argaiz et al. discussed this point [12]. In this way, the pulsatility portal index adds complementary information because it better reflects volemia than the inferior vena cava diameter that better reflects the right/left heart failure. In the study of Argaiz et al. and in our study, the fluid balance was poorly associated with the inferior vena cava diameter and its changes. At baseline, the pulsatility portal index was high and decreased with fluid depletion [27]. The same findings have been demonstrated for the renal venous impedance index. Ter Maaten et al. have previously demonstrated that congestive patients have pulsatile venous renal flows that improved during decongestive therapy, and were normalized to a continuous venous flow in a significant number of patients at hospital discharge [20, 28].

The pulsatility portal index is an indicator of congestion in relation to volemia may be because of the reservoir role of the splanchnic circulation. A recent study on a healthy subject has demonstrated that fluid expansion increased portal blood flow and its pulsatility only in the patient who did not increase their cardiac output [15]. In this sense, portal pulsatility was associated with the predictive of hypervolemia. The splanchnic circulation acts as a blood reservoir that comprises the blood from the intestine, spleen, and liver [29]. This splanchnic blood reservoir can be modulated to increase venous return. In heart failure patients, the splanchnic blood volume is elevated and its capacitance decreases. The pulsatility portal index may indirectly inform on the splanchnic blood flow and the relation between its capacitance and volemia. The observed decrease in pulsatility portal index in both groups (between baseline and 2 hours) may have reflected the venodilatory effect of loop diuretic on splanchnic circulation. But this effect was lower and the pulsatility portal index remained higher in congestive patients because the splanchnic blood volume was probably higher.

On the contrary, the inferior vena cava and the hepatic veins may be more sensitive to heart condition and thoraco-abdominal interactions [30]. Thus, an echographic analysis may reflect these points. It is why we found a low predictability of the VEXUS score. The first step of the VEXUS score is to measure the maximal inferior vena cava diameter that must be higher than 2 cm [11]. But this diameter does not have a strong physiological link with congestion because it depends on several factors [30]. The inferior vena cava diameter did not significantly differ between congestive and non-congestive patients, moreover with underlying mixed heart failure that was the case in our population. In addition, we observed patients with inferior vena cava diameter lower than 2 cm but with venous pulsatility of the portal and/or the vein flows (data not shown).

Most patients’ characteristics were in accordance with guidelines on diuretic-induced fluid depletion. Despite these points, and as previously demonstrated the response to diuretic-induced fluid depletion fluid is highly variable and poorly predictive [31]. We did not demonstrate any association between the amount of fluid depletion and congestion evolution. The quantity of fluid depletion does not make the response because of too much intra-/interindividual variability, while the portal Doppler allows to individually judge the response to treatment. The evaluation should be repeated over time, as suggested by expert consensus for diuresis. The evaluation of the pulsatility portal index over a short period may help to evaluate the capacitance, the volemia of the splanchnic circulation, and their interactions. This evaluation may confirm the effect of fluid depletion on venous congestive parameters. From our exploratory results, the portal pulsatility index may be more sensitive to detect fluid overload than the venous renal flow pattern that could be used to confirm the indication for continuing diuretic depletion. Heart failure and/or hydrosodic overload are not necessary “congestion”, thus Doppler evaluation of the portal and renal flows can bring important clinical elements, even more so during an early evaluation with treatment.

The study has limitations. This is an exploratory, prospective monocentric observational study that includes usual limitations of such designs. Because of inclusion/non-inclusion criteria, we have selected a specific cohort with a high prevalence of cardiovascular comorbidities, with marked signs of left-sided heart failure, mostly fluid unresponsive, and who were included later after admittance to ICU. These points reflect a selected cohort of cardiovascular patients for whom fluid depletion may be necessary, such as in patients with pulmonary weaning failure [32]. Indications for diuretic were left to the physician, but the response to diuretic was evaluated by the evolution of a clinical–biological congestive score [22]. Even though this score was adapted from previous studies, it was in accordance with the literature that demonstrated congestive score being clinically relevant and associated with outcomes [4, 9]. Based on this score, congestive patients had coherent clinical and biological signs of congestion. Because some patients have deceased during the study period, we cannot exclude a competing risk. Most of the patients deceased because of multiple organ failure and/or refractory shock, and they were from the control group. Such an issue can be considered as a failure of treatment and are in line with a pragmatic clinical approach. We did not repeat several and fixed time point echocardiographic measurements to longitudinally evaluate changes until the ICU discharge. The aim was to evaluate the predictability of the pulsatile portal index in the early phase of diuretic treatment.

In conclusion, our study demonstrates that the Doppler study of the portal flow provides information on hemodynamic congestion and fluid overload. The pulsatility portal index and venous renal index predict the appropriate response to diuretic-induced fluid depletion in ICU patients. Further studies integrating these indices may confirm these observations and clarify the usefulness of these indices.

## Supplementary Information

**Additional file 1**: **Table S1**. Hemodynamic and echocardiographic measurements according to congestive response to diuretic treatment (Appropriate fluid depletion vs Control). ^$^*p* < 0.05 comparison between two groups of patient. *p < 0.05 comparison with baseline.

## Data Availability

The datasets used and/or analyzed during the current study are available from the corresponding author upon reasonable request.

## Abbreviations

AUC: Area under the curve
AVA: Aortic valve area
CO: Cardiac output
CW: Continuous wave
HR: Heart rate
HV-D: Sus-hepatic vein diastolic flow velocity
HV-S: Sus-hepatic vein systolic flow velocity
ICU: Intensive care unit
LVEF: Left ventricular ejection fraction
LVOT: Left ventricular outflow tract
PI: Portal pulsatility index
PW: Pulsed wave
ROC: Receiver operating characteristic
RRI: Renal resistive index
RVI: Renal venous impedance index
RVFAC: Right ventricular fraction area change
SAPS II: Simplified acute physiological II score
SV: Stroke volume
TAPSE: Tricuspid annular plane systolic excursion
TR: Tricuspid regurgitation
VEXUS: Venous ultrasound congestion score
Vmax: Maximum velocity
Vmin: Minimum velocity
VTIAo: Aortic velocity–time integral
