# Supplementary material for: Doppler study of portal vein and renal venous velocity predict the appropriate fluid response to diuretic in ICU: a prospective observational echocardiographic evaluation
Source: Crit Care. 2022 Oct 5;26:305. doi: 10.1186/s13054-022-04180-0 (PMC9535945; doi:10.1186/s13054-022-04180-0)
Supplement: Supplementary file 1 — Additional file 1: Table S1. Hemodynamic and echocardiographic measurements according to congestive response to diuretic treatment (Appropriate fluid depletion vs Control). $p < 0.05 comparison between two groups of patient. *p < 0.05 comparison with baseline. [file 13054_2022_4180_MOESM1_ESM.docx]

**Doppler study of portal vein and renal venous velocity predict the appropriate response to diuretic in ICU: a prospective observational echocardiographic evaluation.**

**Definition of the terms used in the study**

- High blood pressure = chronic high blood pressure = high blood pressure is considered to be 140/90 mmHg or 150/90 mmHg or higher if you're over the age of 80 years.
- Diabetes: Insulin-dependent and non-insulin dependent diabetes
- Chronic renal failure: Estimated Glomerular Filtration Rate lower than 60 ml min^-1^ 1.73 m^-2^
- Acute heart failure: rapid or gradual onset of symptoms and/or signs of heart failure, severe enough for the patient to seek urgent medical attention, leading to an unplanned hospital admission or an emergency department visit.
- Cardiogenic shock: clinical signs of hypoperfusion (cold sweated extremities, oliguria, mental confusion, dizziness, narrow pulse pressure), and biochemical manifestations of hypoperfusion (elevated serum creatinine, metabolic acidosis and elevated serum lactate), and/or arterial hypotension.
- Septic shock was defined according to the Third International Consensus Definitions for Sepsis and Septic Shock (Sepsis-3).

**Congestion score**

Congestion score is constructed as follows: pulmonary rales/crackles (graded between 0 (no), 1 (<50% of lung) and 2 (>50% of lung)) [24], peripheral oedema (graded between 0 (no), 1 (ankle), 2 (leg) and 3 (body)), B-lines and/or lung ‘comets’ (graded between 0 (no), 1 (more than 2 area) and 2 (diffuse)), and/or pleural effusion (graded between 0 (no), 1 (unilateral) and 2 (bilateral)) on lung ultrasound, and N-terminal pro B-type natriuretic peptide (NT-proBNP) value over 1500 pg/mL [4,23]. The positive fluid balance was not a criterion in the clinical congestion score calculation. The congestion score ranged from 0 to 10, and a patient with a score ≥ 3 was considered as having significant clinical congestion [4,23]. Appropriate fluid response is defined by a congestion score < 3.

**Table 1.** Hemodynamic and *echocardiographic measurements according* to congestive response to diuretic treatment (*Appropriate fluid depletion vs Control*). ^$^ p<0.05 comparison between two groups of patient. **^*^** p<0.05 comparison with baseline.

|  | **Baseline** | **Two-hours** |
| --- | --- | --- |
| **Hemodynamic parameters**  Heart rate (bpm), mean (SD)  *Appropriate fluid depletion*  *Control*  Mean arterial pressure (mmHg), mean (SD)  *Appropriate fluid depletion*  *Control*  Central venous pressure (mmHg), mean (SD)  *Appropriate fluid depletion*  *Control*  Cardiac index (l min^-1^ m^-2^), mean (SD)  *Appropriate fluid depletion*  *Control*  Diuresis (ml Kg^-1^ h^-1^), median (IQR)  *Appropriate fluid depletion*  *Control* | 83 (16)  80 (18)  80 (11) ^$^  85 (13)  14 (4)  13 (5)  2.7 (0.9) ^$^  2.5 (0.4)  0.5 (0.4-0.7)  0.6 (0.3-0.8) | 82 (14)  82 (16)  79 (12)  85 (13)  12 (4)  12 (5)  2.6 (1)  2.6 (0.8)  1.6 (0.8-2.7) ^*^  1.7 (0.9-2.8) ^*^ |
| **Echocardiographic variables**  LVEF (%), mean (SD)  *Appropriate fluid depletion*  *Control*  Mitral E wave (cm s^-1^), mean (SD)  *Appropriate fluid depletion*  *Control*  Mitral A wave (cm s^-1^), mean (SD)  *Appropriate fluid depletion*  *Control*  Mitral E/A ratio, mean (SD)  *Appropriate fluid depletion*  *Control*  Mitral E/e’ ratio, mean (SD)  *Appropriate fluid depletion*  *Control*  SV change following PLR (%), median (IQR)  *Appropriate fluid depletion*  *Control*  RVFAC (%), mean (SD)  *Appropriate fluid depletion*  *Control*  TAPSE (cm), mean (SD)  *Appropriate fluid depletion*  *Control*  SPAP (mmHg), mean (SD)  *Appropriate fluid depletion*  *Control*  IVC diameter (cm), mean (SD)  *Appropriate fluid depletion*  *Control*  IVC variations (%), median (IQR)  *Appropriate fluid depletion*  *Control*  VTI sus-hepatic S wave (cm s^-1^), median (IQR)  *Appropriate fluid depletion*  *Control*  VTI sus-hepatic D wave (cm s^-1^), median (IQR)  *Appropriate fluid depletion*  *Control*  S/D sus hepatic wave ratio, median (IQR)  *Appropriate fluid depletion*  *Control* | 42 (12)  40 (12)  100 (31)  93 (32)  61 (23)  70 (28)  1.9 (0.8) ^$^  1.4 (0.9)  11 (5)  1 (6)  -3 (-7-5) ^$^  -2 (-4-8)  37 (12)  37 (10)  1.0 (0.3)  1.3 (0.5)  42 (14)  39 (12)  2.2 (0.5)  2.2 (0.4)  6 (2-9)  3 (2-7)  17 (-19-25)  23 (16-33)  30 (24-36)  25 (23-31)  0.6 (-0.5 -1.1)  0.9 (0.6-1.1) | 41 (12)  41 (12)  97 (35)  93 (32)  61(19) ^$^  70 (30)  1.7 (0.9)  1.5 (0.9)  11 (6)  12 (6)  0 (-8-6)  -3 (-10-12)  37 (11)  39 (10)  1.1 (0.4)  1.4 (0.5)  37 (10)  38 (12)  2.1 (0.4)  2.1 (0.4)  7 (3-12)  8 (5-13)  18 (-19-22)  22 (14-30)  30 (23-39) ^$^  20 (18-30)  0.5 (0.4-1.1)  1 (0.6-1.4) |
| Bpm : beats per minute, LVEF: left ventricle ejection fraction, SV: stroke volume, RVFAC: right ventricle fractional area change, TAPSE: tricuspid annular plane systolic excursion, SPAP: systolic pulmonary artery pressure, IVC: inferior vena cava, VTI: velocity time integral, S: systolic, D: diastolic, SD: standard deviation, IQR: interquartile range. | | |
